# Supplementary material for: Sustainable DNA-polysaccharide hydrogels as recyclable bioplastics
Source: Nat Commun. 2025 Aug 12;16:7467. doi: 10.1038/s41467-025-62682-1 (PMC12344013; doi:10.1038/s41467-025-62682-1)
Supplement: Supplementary file 1 — Supplementary Information [file 41467_2025_62682_MOESM1_ESM.pdf]

Supplementary Information

Sustainable DNA-Polysaccharide Hydrogels as Recyclable Bioplastics

Yujie Ke,[1,2+] Kai Lan,[3,+] Jing Yi Wong,[1,4] Hongfang Lu,[1,5] Shujun Gao,[6] Keunhyuk Ryu,[7] Feng Chen,[1,5] Wei Wei Loh,[1] Zhili Dong,[7] Jason Y. C. Lim,[1,8] Zhaogang Dong,[1,9] Xi Chen,[2,*] Itamar Willner,[10,*] Yuwei Hu[1,*]

1. Institute of Materials Research and Engineering (IMRE), Agency for Science, Technology and Research (A*STAR), 2 Fusionopolis Way, Innovis #08-03, Singapore 138634, Republic of Singapore.

2. School of Interdisciplinary Studies, Lingnan University, Tuen Mun, Hong Kong SAR, China.

3. Department of Forest Biomaterials, North Carolina State University, 2820 Faucette Drive, Raleigh, North Carolina, 27695, USA.

4. Department of Biomedical Engineering, National University of Singapore, 4 Engineering Drive 3, Singapore 117543, Republic of Singapore.

5. Institute of Molecular and Cell Biology, Agency for Science Technology and Research (A*STAR), 61 Biopolis Drive, The Proteos, Singapore 138673, Republic of Singapore.

6. National Neuroscience Institute, 11 Jln Tan Tock Seng, Singapore, 308433, Republic of Singapore.

7. School of Materials Science and Engineering, Nanyang Technological University, Singapore 639798, Republic of Singapore.

8. Department of Materials Science and Engineering, National University of Singapore, 9 Engineering Drive 1, Singapore 117576, Republic of Singapore.

9. Science, Mathematics, and Technology (SMT), Singapore University of Technology and Design (SUTD), 8 Somapah Road, Singapore 487372, Republic of Singapore.

10. Institute of Chemistry, The Center for Nanoscience and Nanotechnology, The Hebrew University of Jerusalem, Jerusalem 91904, Israel.

[+] These authors contributed equally (Y.K., K.L.).

* Corresponding authors: X. Chen (chenxi@ln.edu.hk), I. Willner (itamar.willner@mail.huji.ac.il), Y. Hu (ywhu@imre.a-star.edu.sg)

This PDF file includes:

Supplementary Figs. S1 to S31

Supplementary Note S1

Supplementary Table S1 to S4

Supplementary References

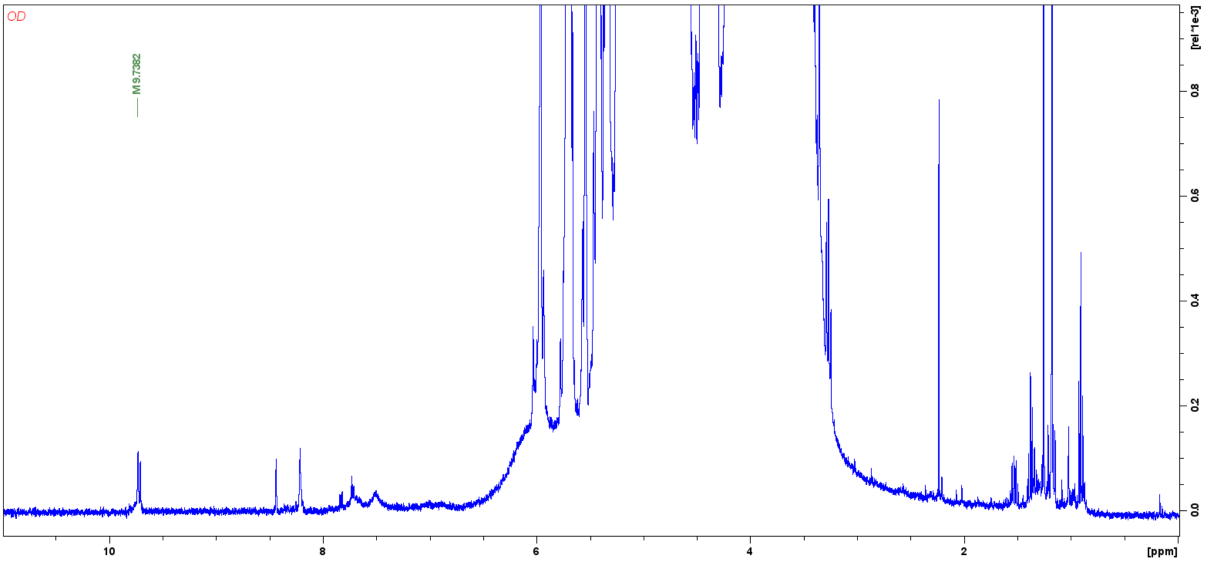

Fig. S1 ^{1}H NMR spectrum of oxidized Dex, exhibiting the aldehyde signal at ~δ 9.7 ppm (D_2O, 400 MHz).

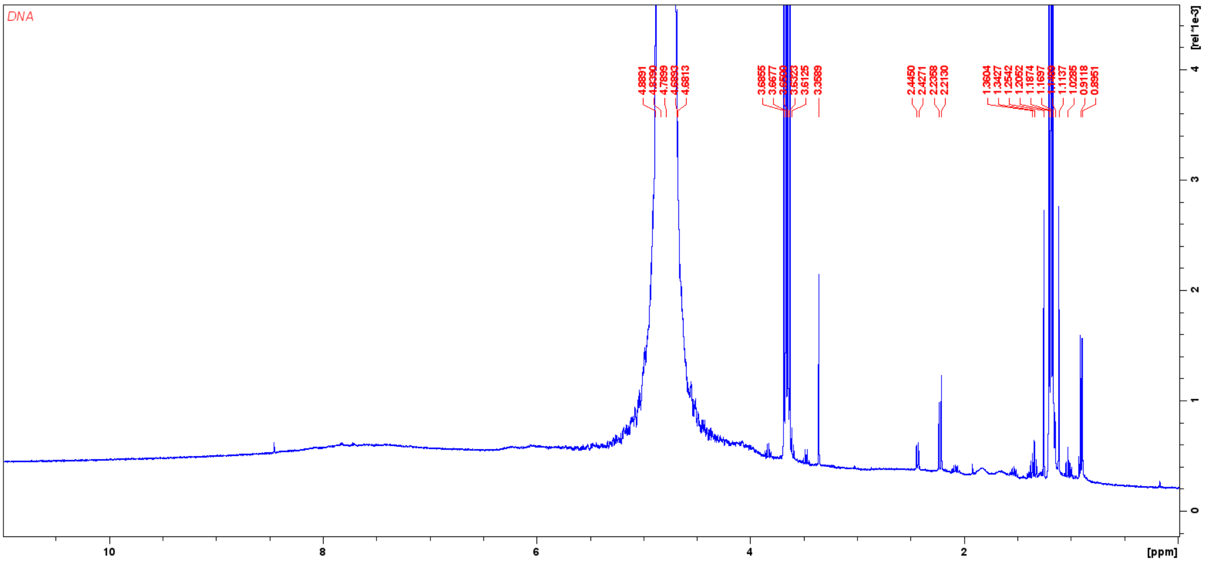

Fig. S2 ^{1}H NMR spectrum of DNA (D_2O, 400 MHz).

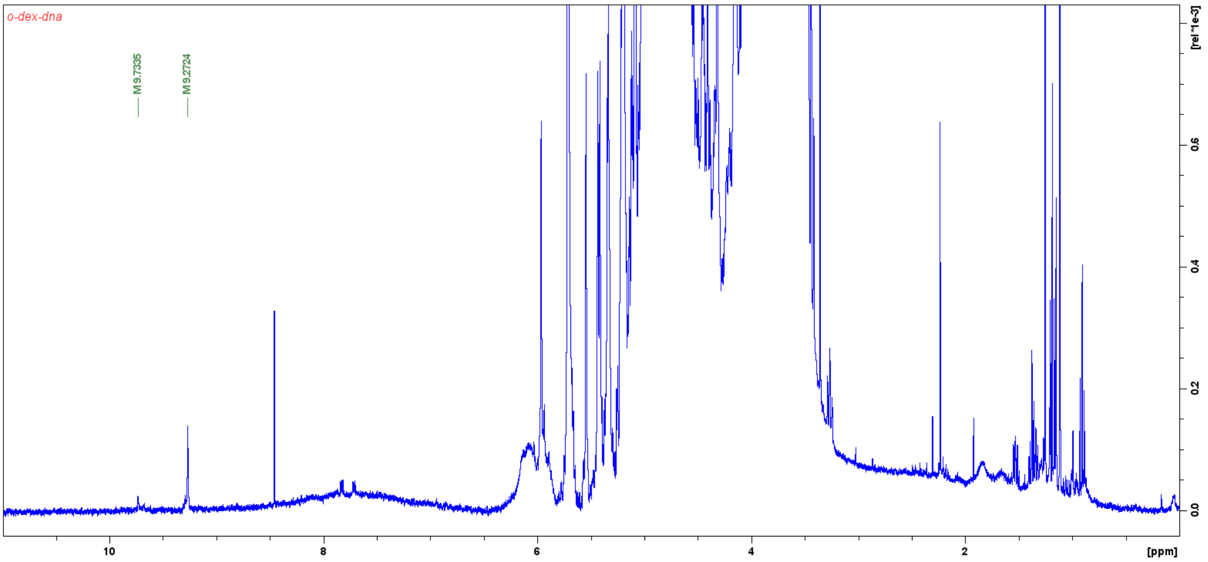

Fig. S3 ^{1}H NMR spectrum of Dex-DNA, exhibiting additional imine (RN=CHR') signals at δ 9.2–9.3 ppm (D_2O, 400 MHz).

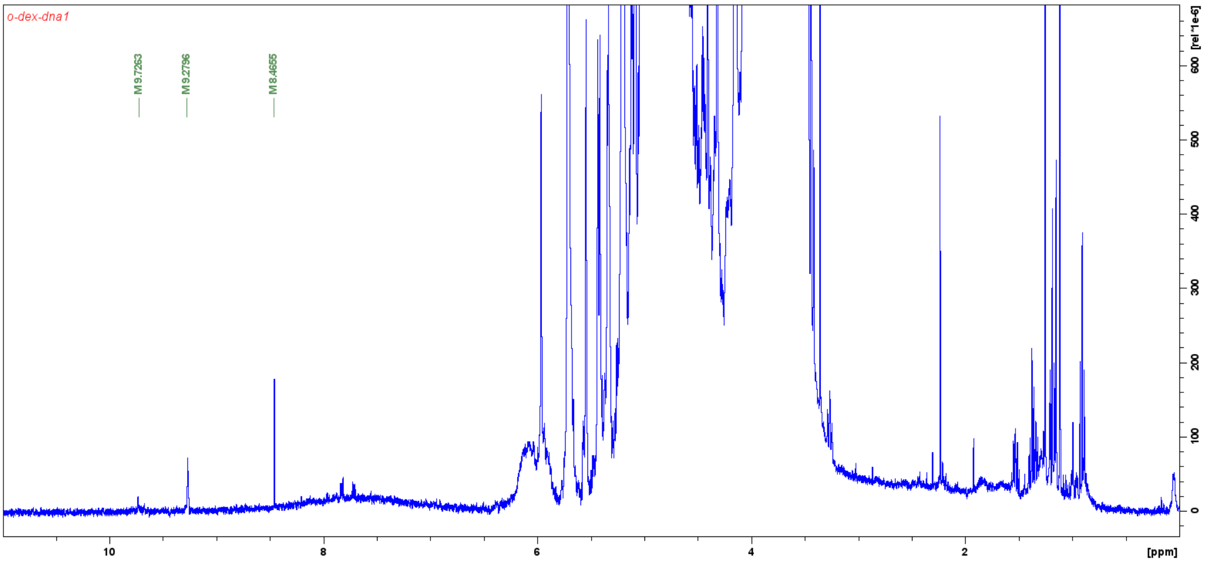

Fig. S4 ^{1}H NMR spectrum of recycled Dex-DNA, exhibiting additional imine (RN=CHR') signals at δ 9.2–9.3 ppm (D_2O, 400 MHz).

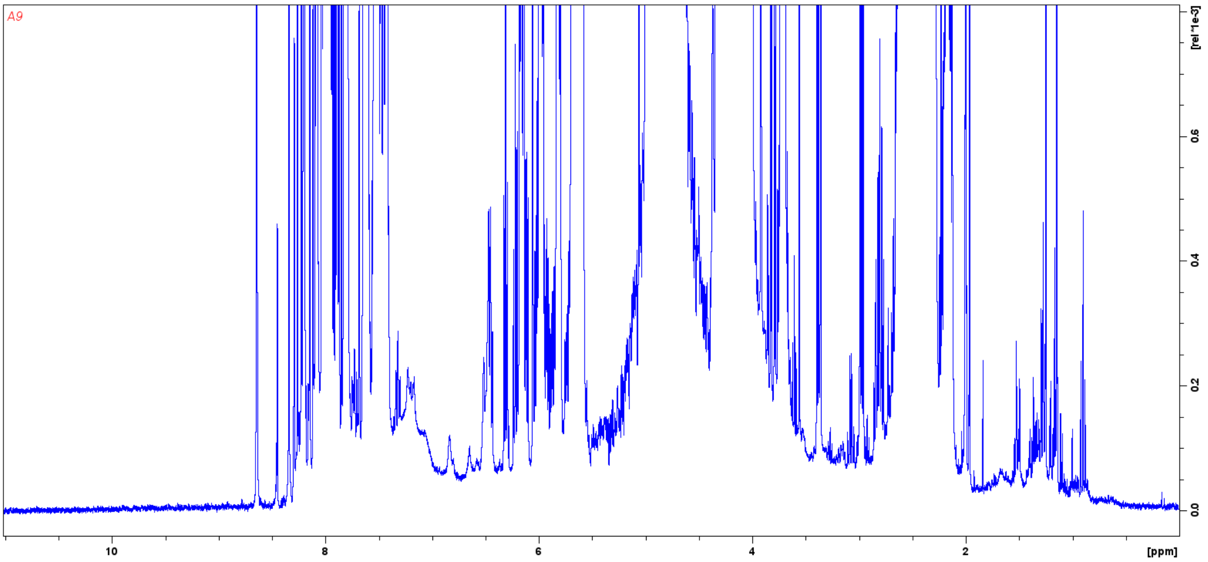

Fig. S5 ^{1}H NMR spectrum of oligo A9 (5'-AAA AAA AAA-3') (D_2O, 400 MHz).

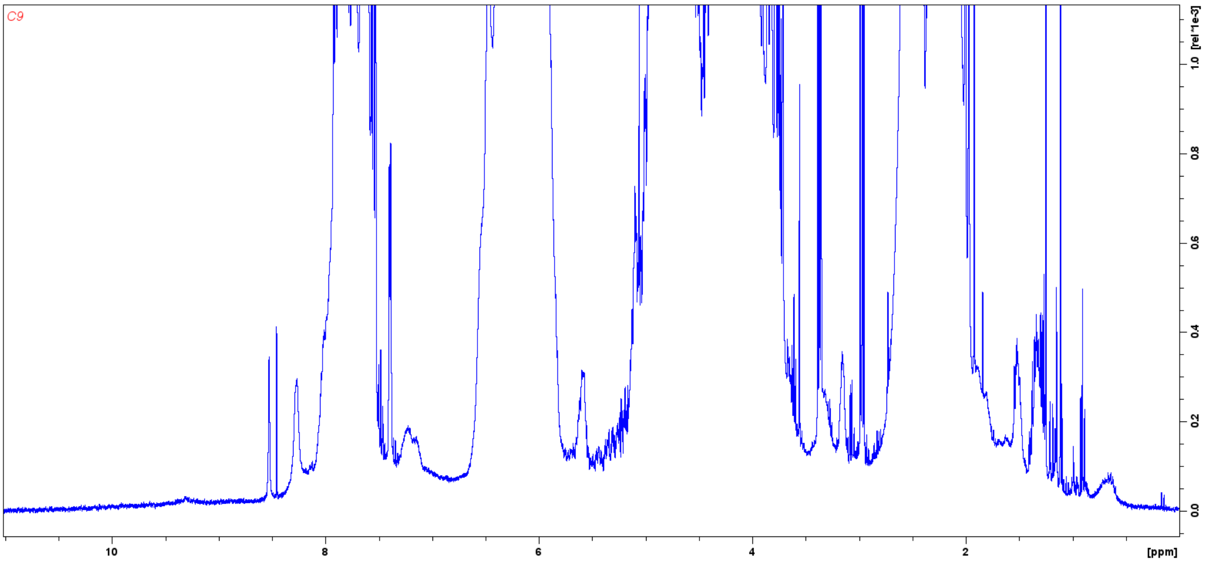

Fig. S6 ^{1}H NMR spectrum of oligo C9 (5'-CCC CCC CCC-3') (D_2O, 400 MHz).

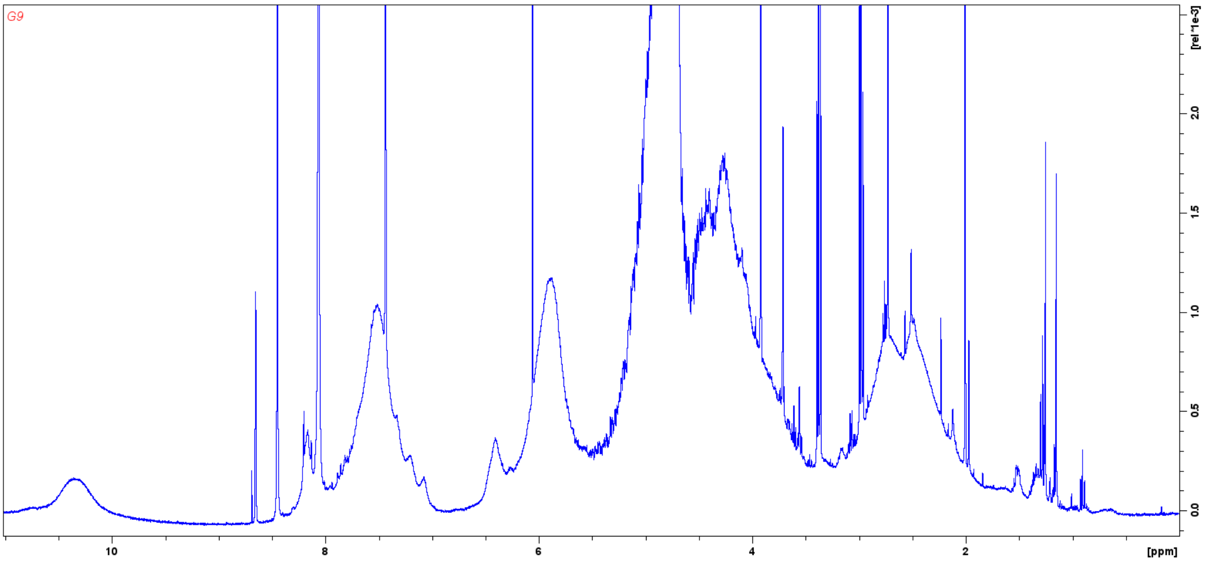

Fig. S7 ^{1}H NMR spectrum of oligo G9 (5’-GGG GGG GGG-3’) (D_2O, 400 MHz).

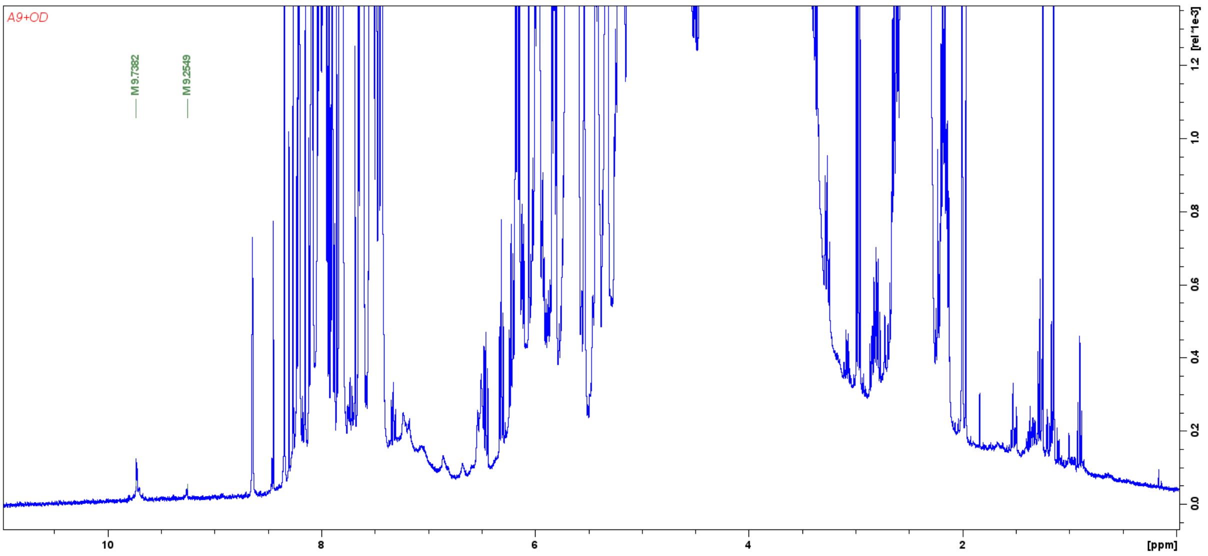

Fig. S8 ^{1}H NMR spectrum of Dex-A9. In comparison to Dex (Fig. S1) and A9 (Fig. S5), an additional signal of imine at ~δ 9.25 ppm is observed (D_2O, 400 MHz).

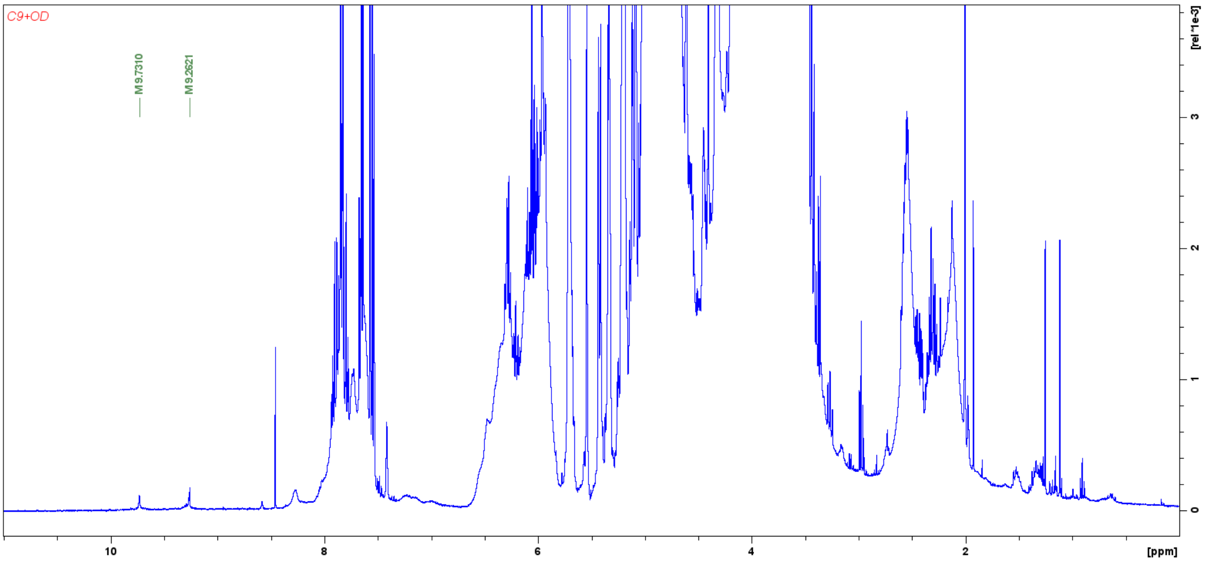

Fig. S9 ^{1}H NMR spectrum of Dex-C9. In comparison to Dex (Fig. S1) and C9 (Fig. S6), an additional imine signal at ~δ 9.26 ppm is observed (D_2O, 400 MHz).

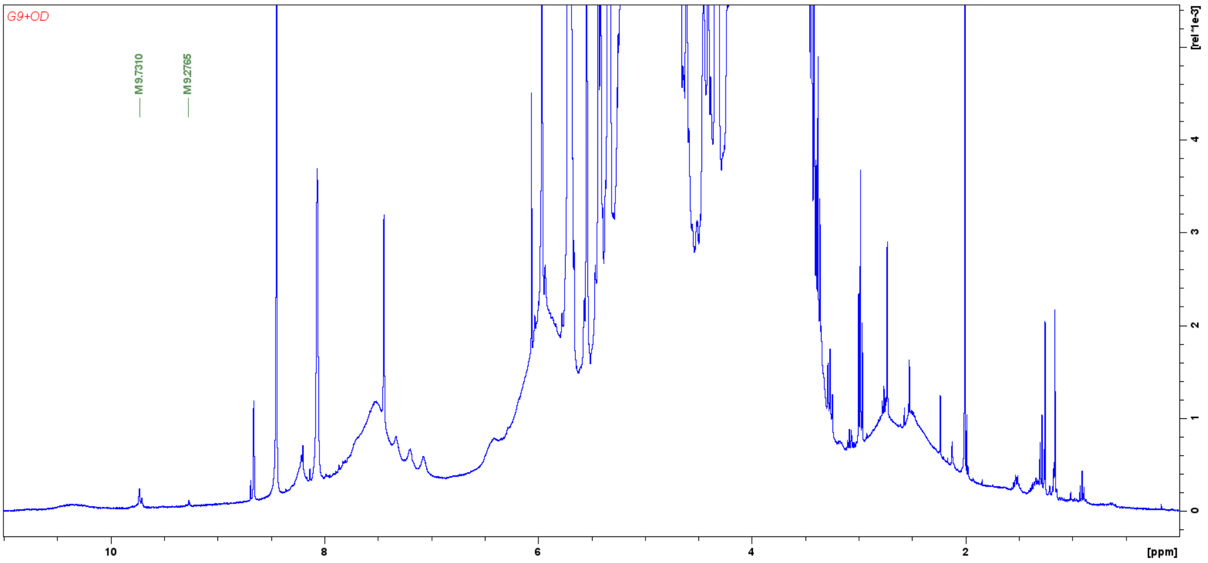

Fig. S10 ^{1}H NMR spectrum of Dex-G9. In comparison to Dex (Fig. S1) and G9 (Fig. S7), an additional imine signal at ~δ 9.27 ppm is observed (D_2O, 400 MHz).

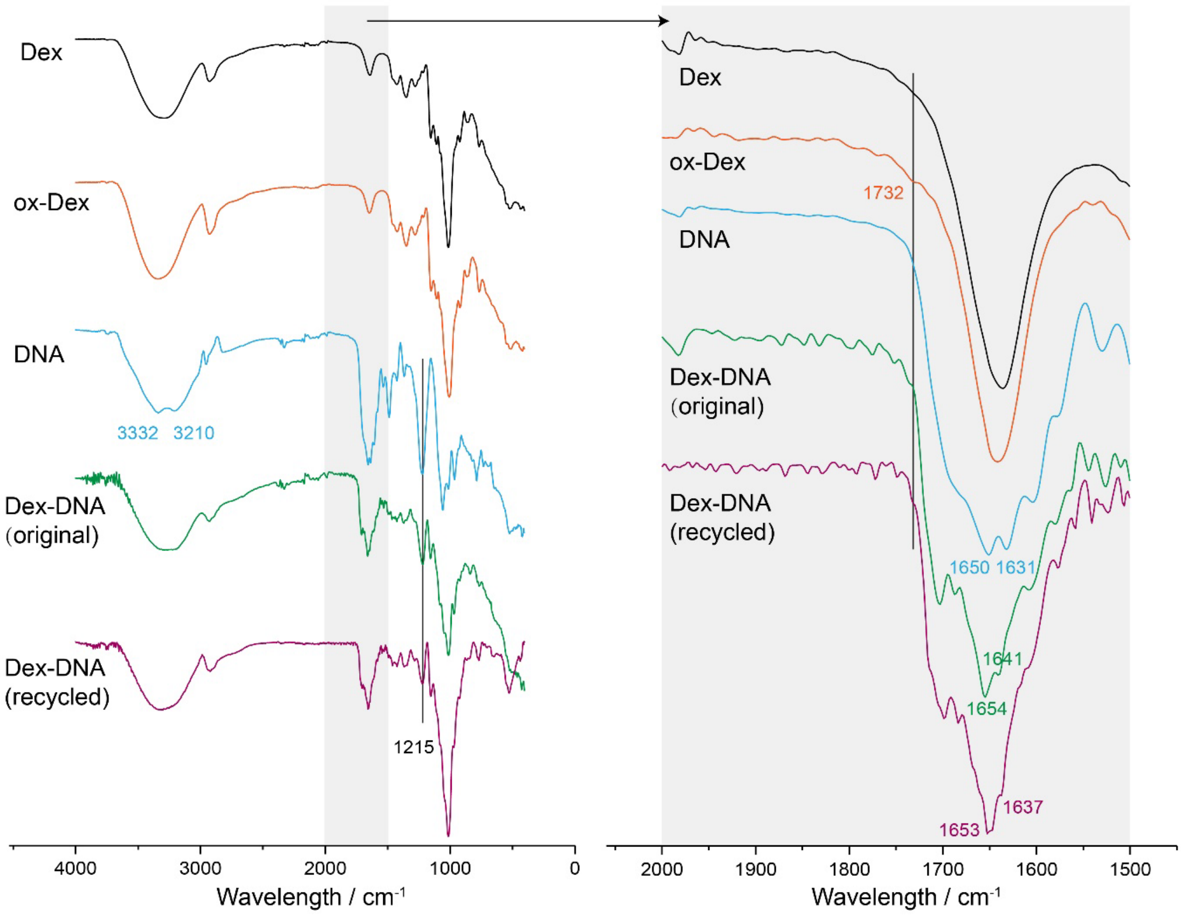

Fig. S11 FTIR spectra of and analysis for the Dex, ox-Dex, DNA, Dex-DNA, and recycled Dex-DNA.

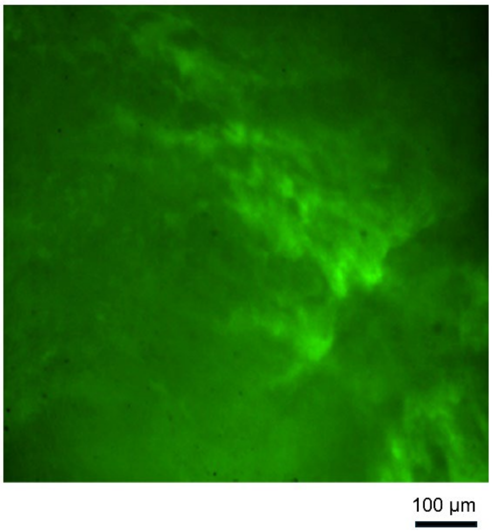

Fig. S12 Fluorescent image of the Dex-DNA hydrogels stained with SYBR Green I.

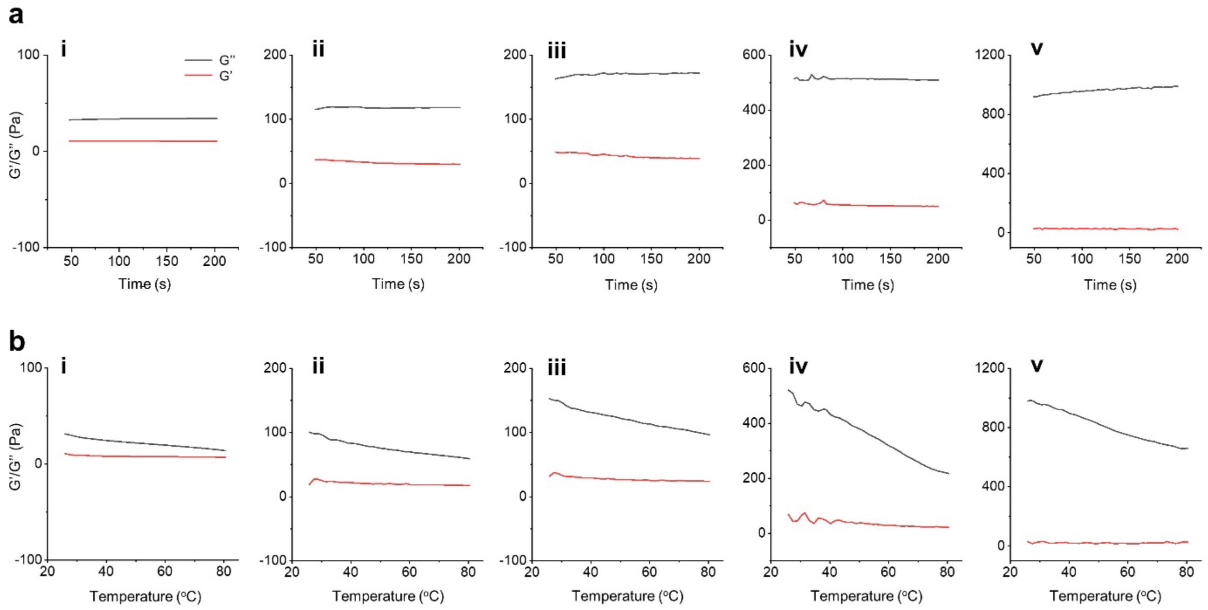

Fig. S13 Rheology results of Dex-DNA hydrogels with a fixed DNA content of 5 wt.% and a variable Dex of 2, 5, 10, 15, and 20 wt. % from left to right. (a) The G′/G″ under the change with time. (b) The G′/G″ under the change with temperature.

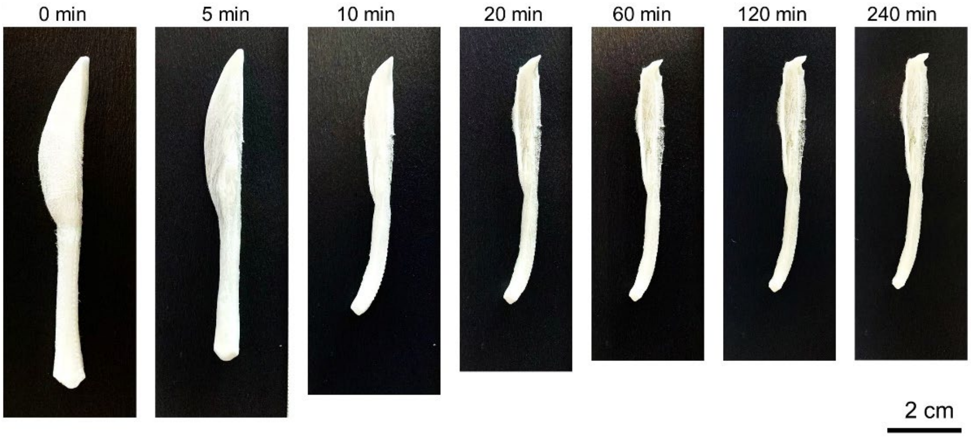

Fig. S14 Photographs of the 2%Dex-2%DNA composite showing the shrinkage processes under atmospheric conditions after taking it out from the freeze dryer.

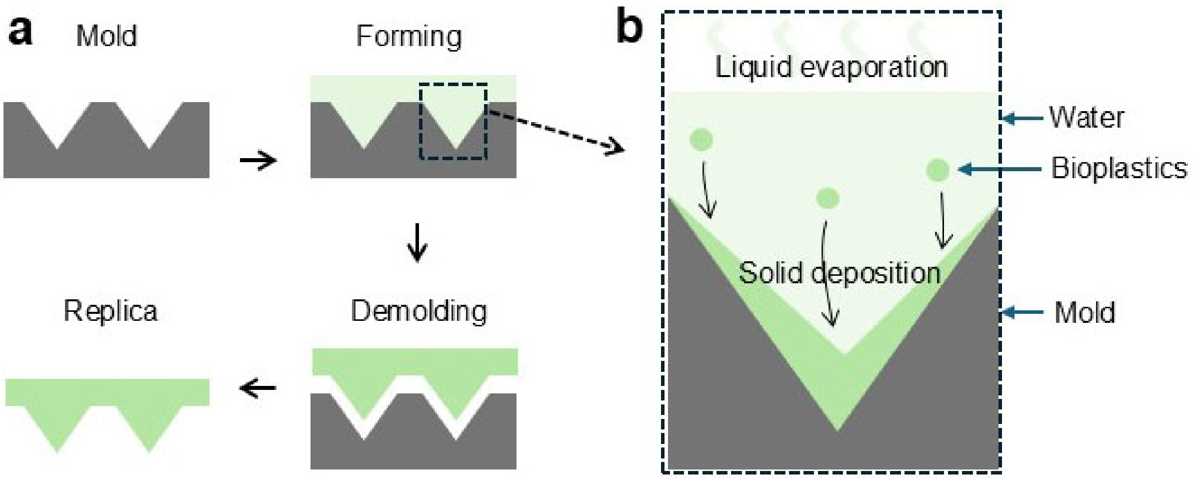

Fig. S15 (a) Schematics of the molding process to produce the bioplastics with high precision through an air-drying process under room temperature. (b) Illustration of the formation of bioplastics in nano-/micro-scale through a deposition in liquid phase assisted by the liquid evaporation.

	original	1st recycled	2nd recycled
WVTR (g/m²·day)	480.0	497.8	487.6
P (g·mm/m²·day·*k*Pa)	195.5	202.7	198.6

Fig. S16 The WVTR and peameability (*P*) values of the original and recycled bioplastics.

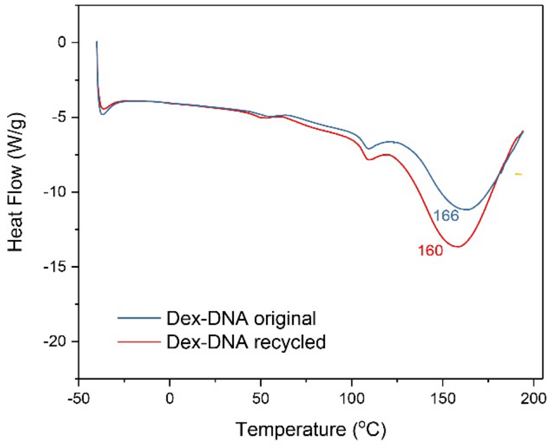

Fig. S17 The DSC analysis the original and recycled bioplastics.

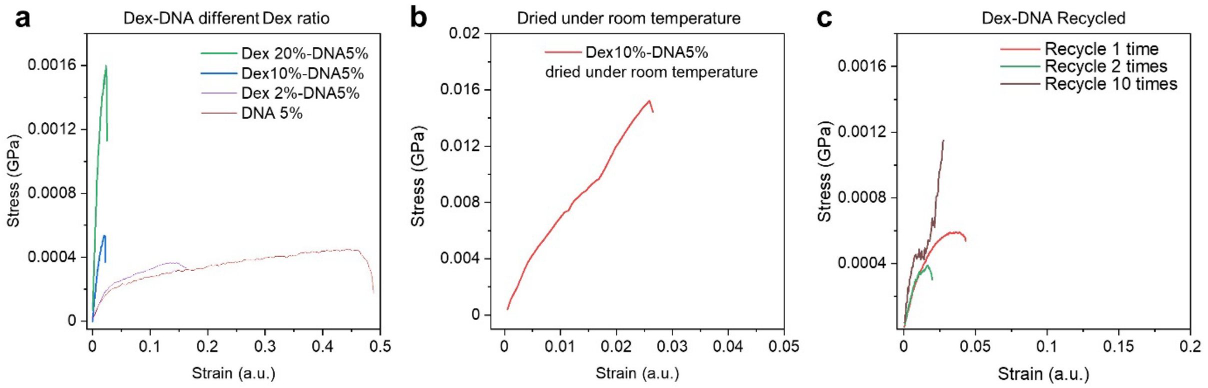

Fig. S18 The stress-strain curves of the (a) Dex-DNA solid composites produced via the hydrogels with Dex content ranging from 0% to 20%, (b) 10%Dex-5%DNA solid prepared via a direct drying process at room temperature, and (c) recycled 10%Dex-5%DNA composites after1, 2, and 10 recycling cycles.

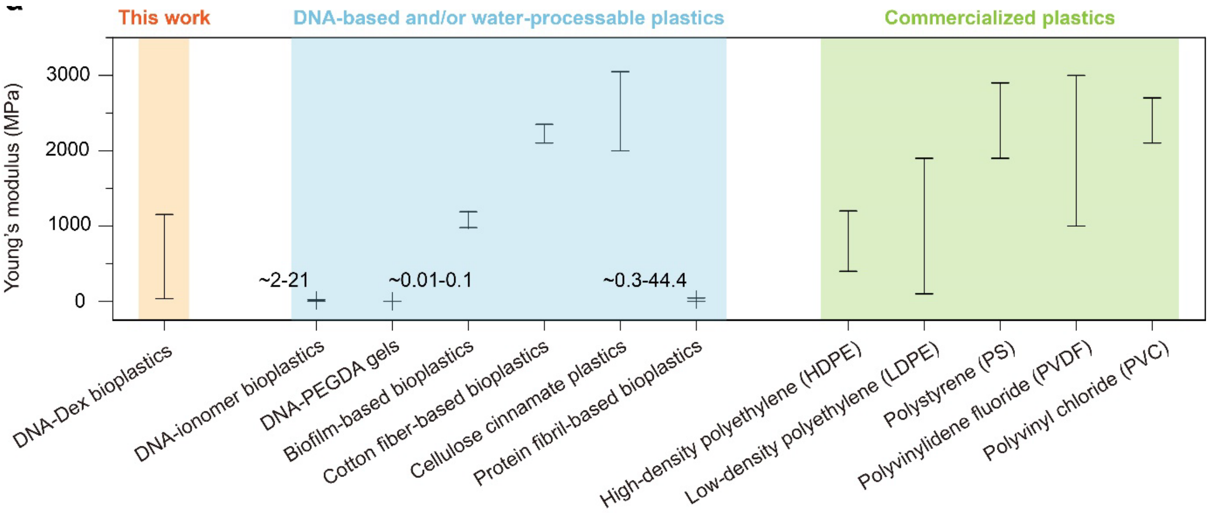

Fig. S19 Comparison of Young's modulus of the Dex-DNA bioplastics in this work with reported DNA-based and/or water-processable plastics in literature and several typical commercial plastics. These reported plastics included DNA-ionomer bioplastics[1], DNA-PEDGA gels[2], biofilm-based bioplastics[3], cotton fiber-based bioplastics[4], cellulose cinnamater bioplastics[5], and protein fibril-based bioplastics[6]. The data of these commercialized plastics are from Materials Data resources provided by Matmake website[7].

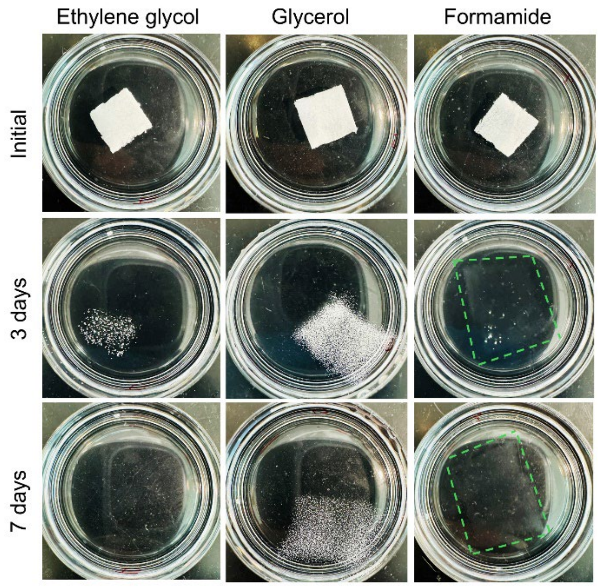

Fig. S20 Photographs of the chemical resistance test for 10%Dex-5%DNA bioplastics in ethylene glycol, glycerol, and formamide. Initial dimension 1×1 cm.

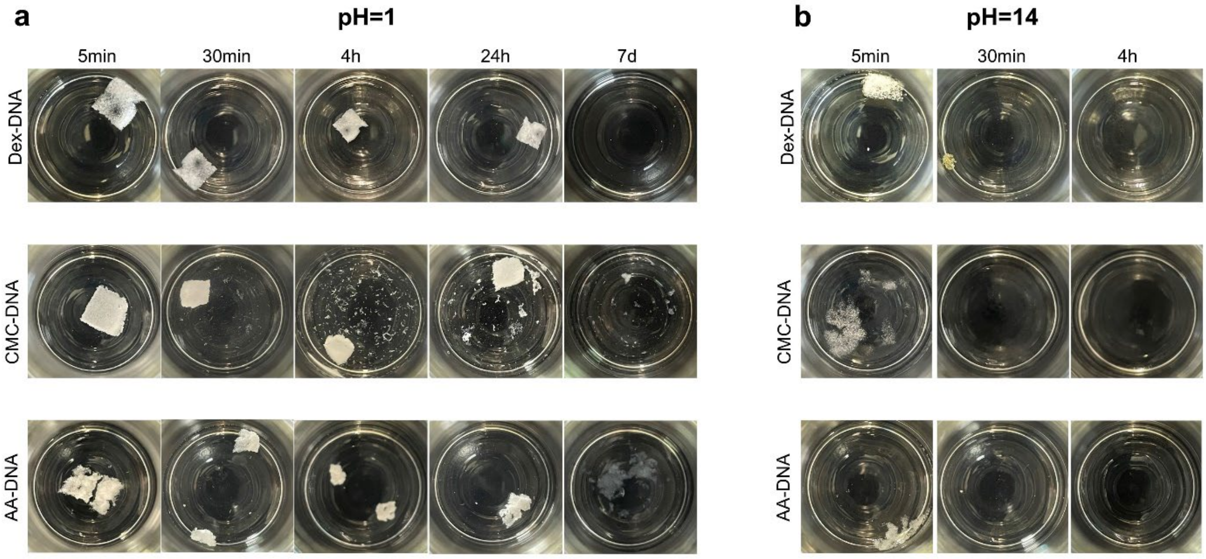

Fig. S21 Photographs of the three bioplastic pieces soaking in (a) pH 1 and (b) pH 14 solutions, where all three bioplastics significantly dissolved in pH 1 and pH 14 solutions after 7 days and 4 hours, respectively. Initial dimension 1×1 cm.

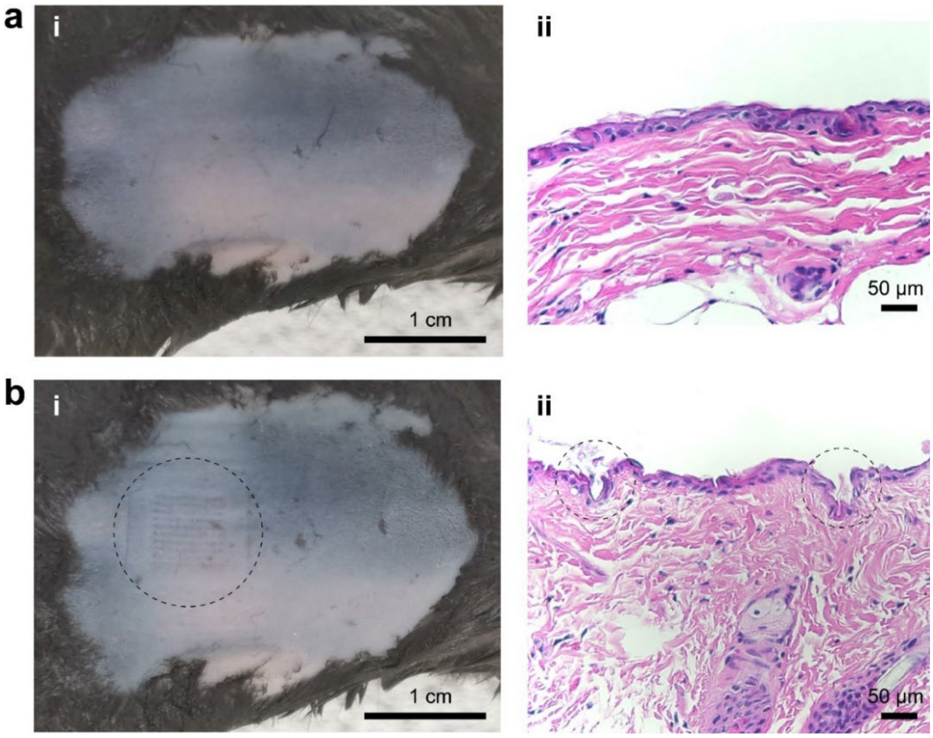

Fig. S22 The stratum corneum before (a) and after (b) penetration by the bioplastic microneedle arrays. (a i and b i) Images of the applied mouse tissue. (a ii and b ii) Representative microscopy images of H&E stained stratum corneum cross sections.

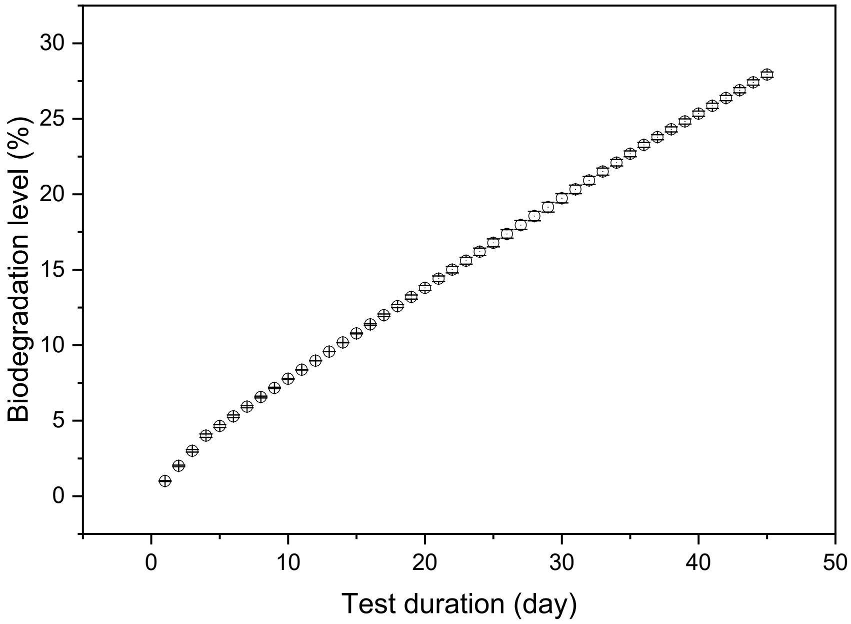

Fig. S23 Biodegradation test result using the ASTM standard method. Continuous biodegradation was observed over the 45-day test period, reaching approximately 28% degradation by day 45. Data are presented as mean ± standard deviation (n = 3).

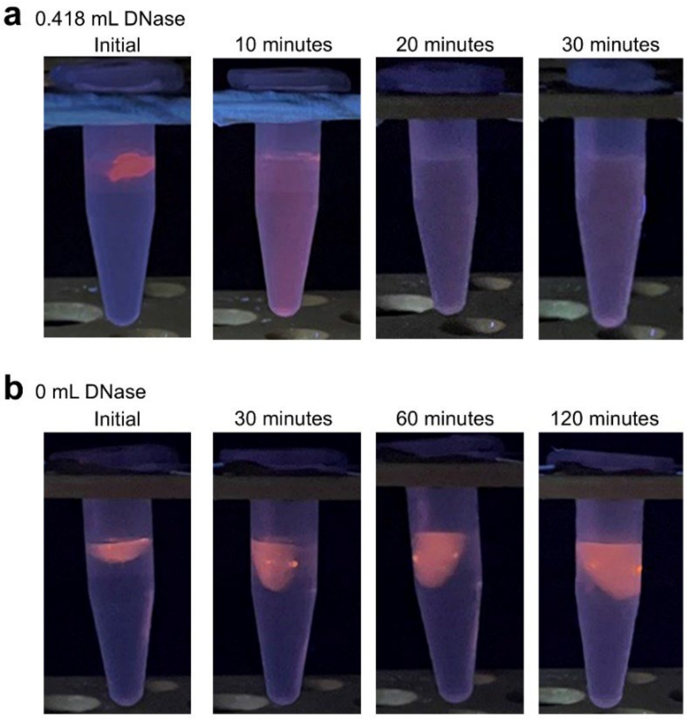

Fig. S24 (a) Photographs of the facilitated biodegradation process of the bioplastics by mixing in a high-concentration DNase I solution. The bioplastics were stained with GelRed for better illustration. (b) The control sample test with the bioplastics sample soaking in water without the addition of DNase I.

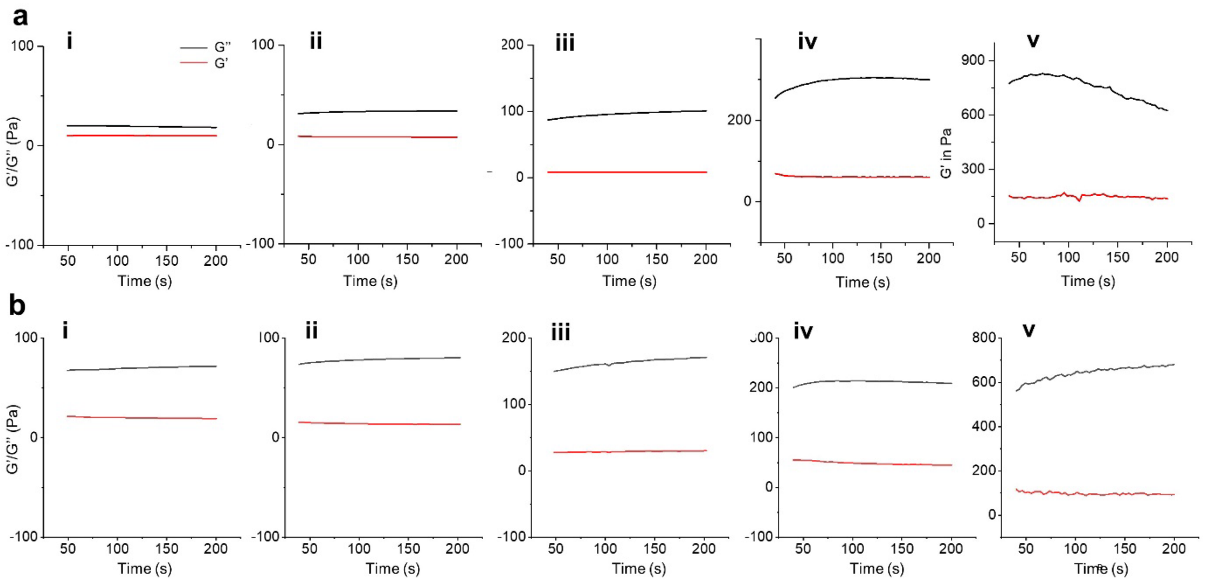

Fig. S25 Rheology results of (a) AA-DNA and (b) CMC-DNA hydrogels with a fixed DNA content of 5 wt.% and a variable AA or CMC content of 2, 5, 10, 15, and 20 wt. % from left to right.

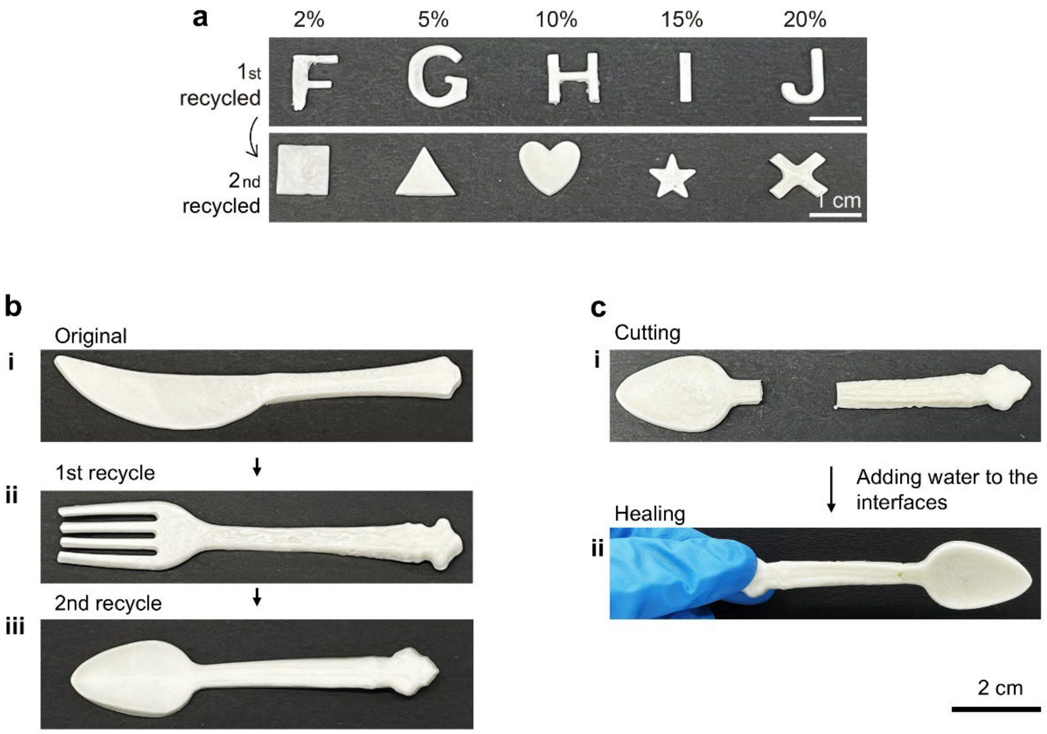

Fig. S26 (a) Photo images of the 1st and 2nd recycled AA-5%DNA samples produced using the hydrogels with AA contents ranging from 2% to 20%. (b) Produced 10%AA-5%DNA bioplastic products and recycled products. (c) Photographs to illustrate the aqua-healing process of a 10%AA-5%DNA bioplastic product.

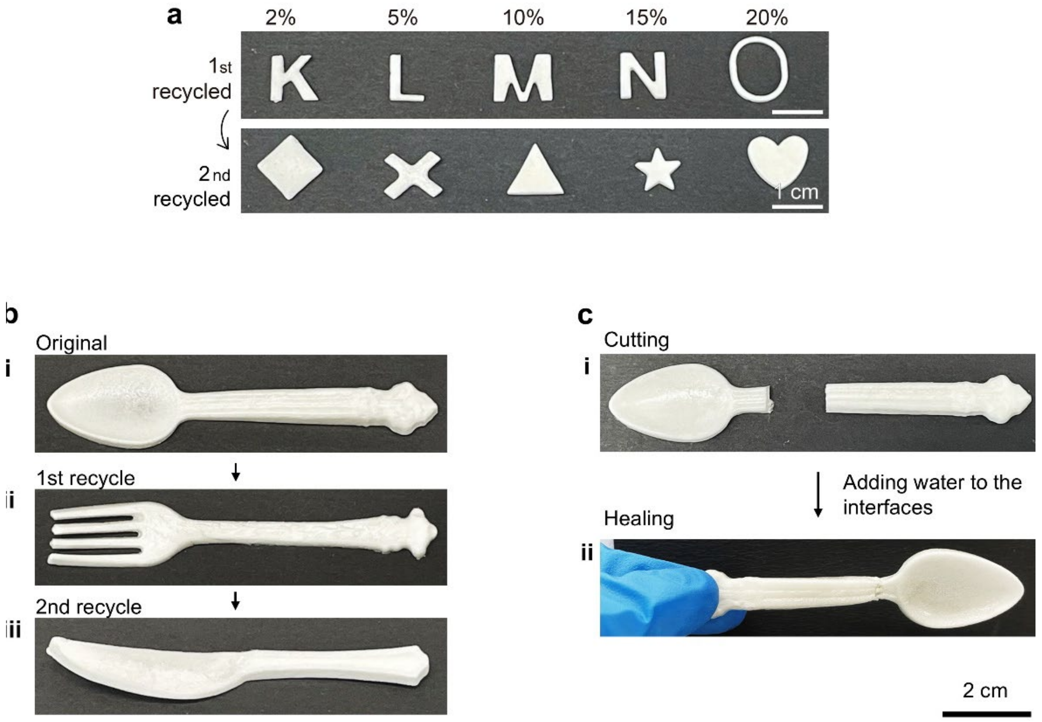

Fig. S27 (a) Photographs of the 1st and 2nd recycled CMC-5%DNA samples produced using the hydrogels with CMC content ranging from 2% to 20%. (b) Produced 10%CMC-5%DNA bioplastic products and their recycled products. (c) Photographs to illustrate the aqua-healing process of a 10%CMC-5%DNA bioplastic product.

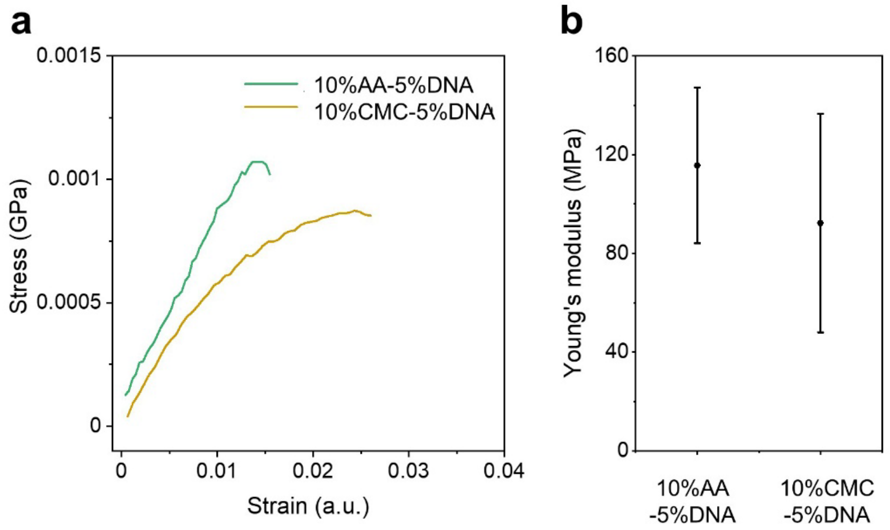

Fig. S28 (a) The stress-strain curves of the 10%AA-5%DNA and 10%CMC-5%DNA solid composites and (b) their Young's modulus. Data are presented as mean ± standard deviation (n = 3).

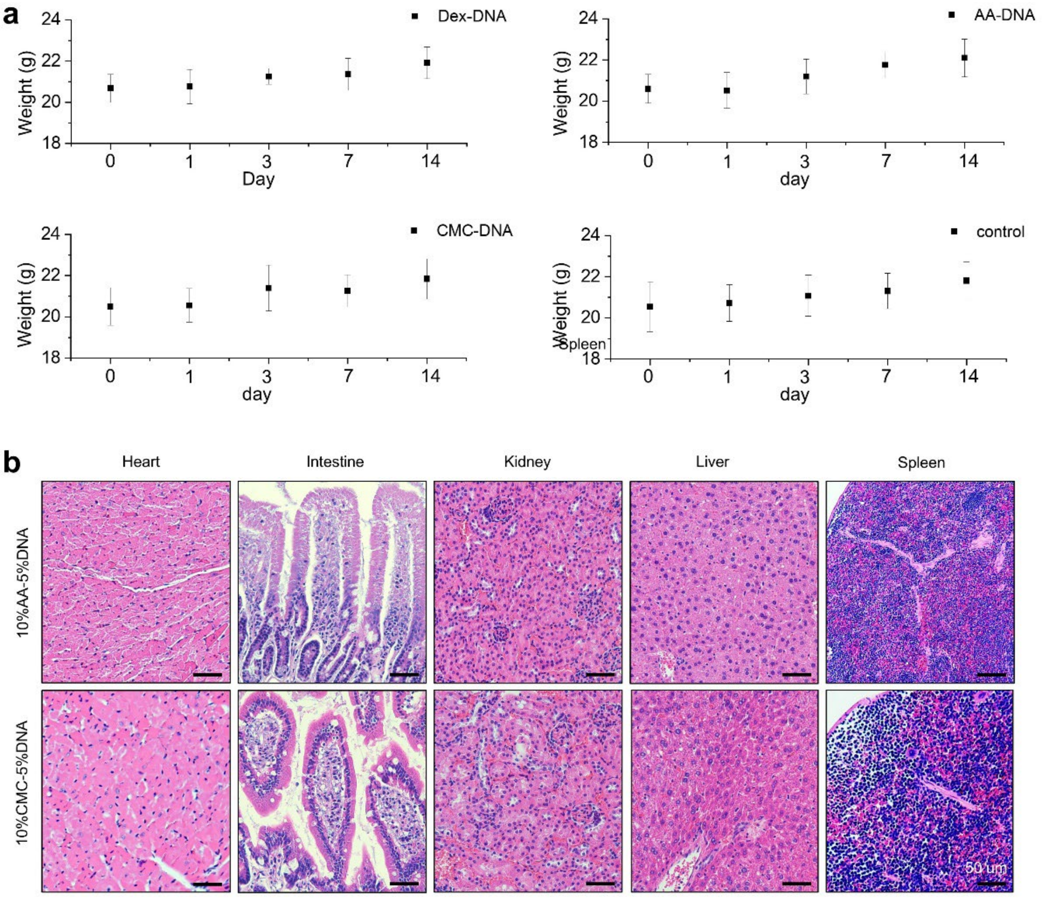

Fig. S29 (a) Mice weight analysis after taking the Dex-DNA, AA-DNA, and CMC-DNA composites, as well as the control sample. Data are presented as mean ± standard deviation (n = 3). (b) Representative microscopy images of stained mice organs sections in 14 days after digesting the AA-DNA and CMC-DNA. Organ examination includes heart, intestine, kidney, liver, and spleen.

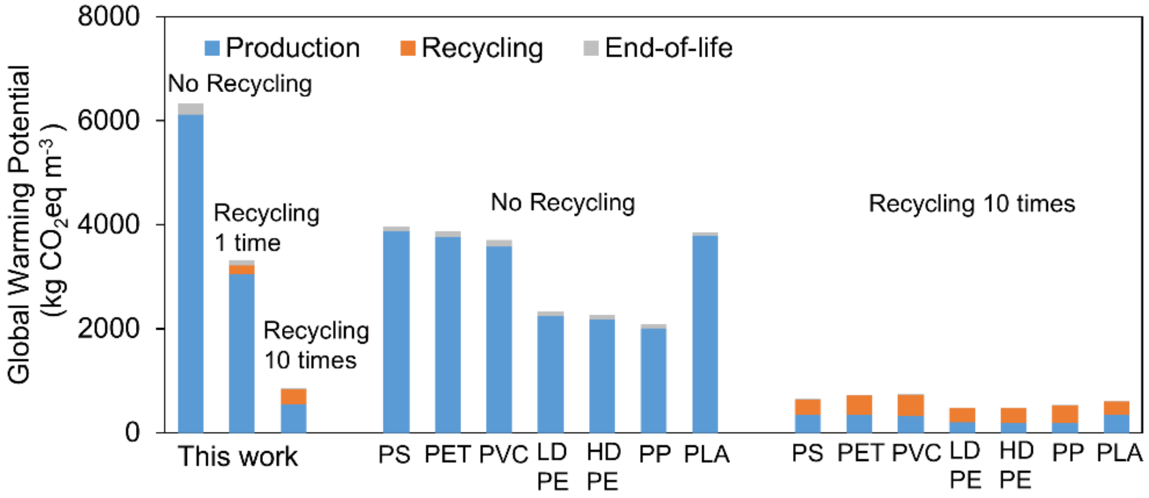

Fig. S30 Comparison of the global warming potential per use cycle across different recycling times for the bioplastics versus commercial plastics, including PS (polystyrene), PET (polyethylene terephthalate), PVC (polyvinyl chloride), LDPE (low-density polyethylene), HDPE (high-density polyethylene), PP (polypropylene), and PLA (polylactic acid).

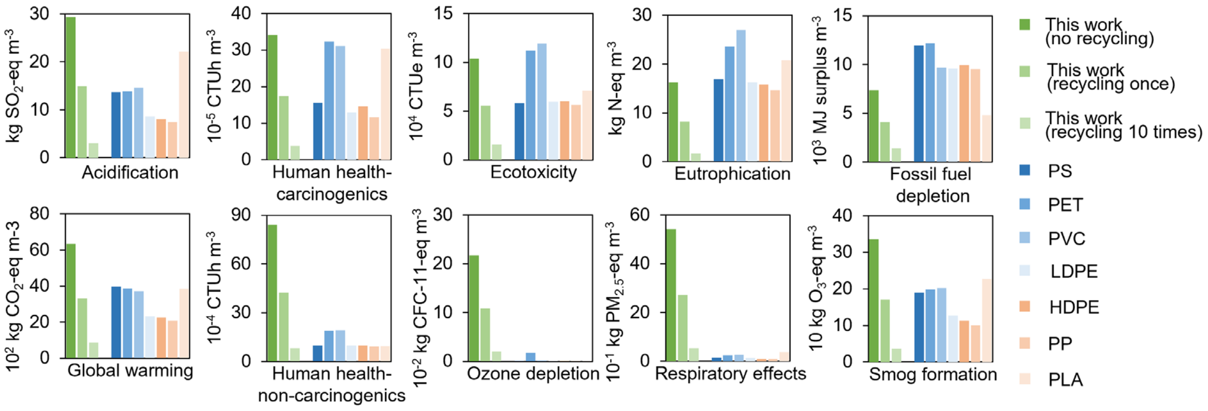

Fig. S31 Environmental impact comparison between bioplastics and commercial plastics across multiple metrics. The commercial plastics include PS (polystyrene), PET (polyethylene terephthalate), PVC (polyvinyl chloride), LDPE (low-density polyethylene), HDPE (high-density polyethylene), PP (polypropylene), and PLA (polylactic acid). CTUe represents Comparative Toxicity Unit for ecotoxicity; CTUh represents Comparative Toxicity Unit for human health; CFC-11-eq represents chlorofluorocarbon equivalent for ozone depletion; PM2.5-eq represents particulate matter 2.5 equivalent for air pollution.

Note S1. Life-cycle environmental impacts of bioplastics.

The life cycle assessment (LCA) is conducted following the ISO standard series 14040[8]. The life cycle inventory (LCI) data of the upstream burdens of producing materials, chemicals, electricity, fuels, and water are collected from the ecoinvent database[9] and literature. The LCI data for producing and recycling bioplastics are shown in Supplementary Table S1. The Dex production was estimated based on the mature technology of using sucrose and dextransucrase and precipitating via ethanol[10-13]. The LCI data for DNA production was estimated based on the studies by Allen et al.[14] in *Nature Protocols* and Reads[15]. The ground plant leaf tissues were mixed with extraction buffer that contained 0.1 M Tris-HCl (tris(hydroxymethyl)aminomethane hydrochloride), 1.0 M NaCl, 0.02 EDTA (ethylenediaminetetraacetic acid), and 2% CTAB (cetyltrimethylammonium bromide)[14,15]. Then the solution was extracted with an equal volume of chloroform: isoamyl alcohol (24:1, v/v) and centrifuged to separate the aqueous phase[14,15]. The DNA then was precipitated in isopropanol and washed with 70% ethanol[14,15]. See Supplementary Table S2 for detailed inputs and outputs. The production of Tris follows the patent by Bourguignon et al..[16]. The CTAB can be commercially synthesized by cetyl(hexadecyl) bromide and trimethylamine[17]. For sodium iodate, the production follows the patent by Liu and Song that can produce sodium iodate at a large scale[18]. The method uses $NaClO_3$, iodine, water (11 : 6 : 3, mole ratio), and strong nitric acid to control the pH <1. After 1 h, NaOH solution is added to derive the sodium iodate crystal. The vented chlorine gas is captured by NaOH solution[18]. In this study, the environmental impacts of commercialized plastics are based on the ecoinvent database, including polystyrene (PS), polyethylene terephthalate (PET), polyvinyl chloride (PVC), low-density polyethylene (LDPE), high-density polyethylene (HDPE), polypropylene (PP), and polylactic acid (PLA). The end-of-life of bioplastics in this study and PLA is assumed to be industrial composting, while other plastics are assumed to be landfilled. Hence, the biogenic carbon in the bioplastics and PLA will be fully released back into the atmosphere in the end-of-life stage[9]. The environmental impact assessment method adopted by TRACI 2.1 developed by U.S. Environmental Protection Agency[19] with Global Warming Potential 100 years factors recently released by the Intergovernmental Panel on Climate Change[20].

Table S1. The contribution analysis of life-cycle environmental impacts for bioplastics with recycling 10 times.

**Environmental Impact Category**	Dextran	DNA	$NaIO_4$	Electricity	Water
Acidification	12.1%	68.7%	1.8%	16.2%	0.0%
Carcinogenics	9.8%	63.8%	3.2%	22.7%	0.1%
Ecotoxicity	8.8%	39.8%	0.8%	50.4%	0.0%
Eutrophication	20.0%	65.5%	2.1%	12.1%	0.0%
Fossil fuel depletion	2.5%	30.7%	1.3%	65.4%	0.0%
Global warming	4.3%	50.0%	1.7%	41.6%	0.0%
Non carcinogenics	9.8%	83.4%	0.3%	6.5%	0.0%
Ozone depletion	0.0%	99.9%	0.0%	0.0%	0.0%
Respiratory effects	9.9%	78.0%	2.3%	9.4%	0.1%
Smog formation	9.3%	68.7%	1.9%	19.9%	0.0%

Table S2. Estimated cost calculation of DNA-polysaccharide bioplastics

Raw Materials	Price	Price Reference	Price per 1 Gram	Price per 1 Gram of Bioplastics
Biomass DNA*	8.4 USD/ 1 G	References[1,21]	8.4 USD	2.8 USD
Dex	1673 USD/500 G	Sigma-Aldrich	3.3 USD	2.2 USD
AA	464 USD/1 KG	Sigma-Aldrich	0.5 USD	0.3 USD
CMC	165 USD/1 KG	Sigma-Aldrich	0.17 USD	0.1 USD
$NaIO_4$	3016 USD/2.5 KG	Sigma-Aldrich	1.2 USD	0.2 USD

Bioplastics	Estimated cost per 1 Gram of Bioplastics
10%Dex-5%DNA*	5.2 USD
10%AA-5%DNA*	3.3 USD
10%CMC-5%DNA*	3.1 USD

Table S3. The materials and energy consumption of producing and recycling 1 m^3 bioplastics.

**Item**	**Value**	**Unit**
Production		
Dextran	100.05	kg
DNA	49.95	kg
Sodium Iodate	26.40	kg
Water	4100	kg
Electricity for mixing	507.0	kWh
Electricity for centrifuging	487.5	kWh
Electricity for freezing and drying	180.1	kWh
Transportation	4.5	t·km
Recycling		
Electricity	630.1	kWh
Water	850.5	kg
Transportation	4.5	t·km

Table S4. The inputs and outputs of producing chemicals and materials.

	Item	Value	Unit
Dextran			
Input	sucrose	2.71	kg
	water	19.86	kg
	enzyme	0.11	kg
	ethanol	0.18	kg
	electricity	0.10	kWh
	wood pellets	2.17	kg
Output	dextran	1.00	kg
	wastewater	21.86	kg
DNA			
Input	plant leaves	83.3	kg
	tris	0.05	kg
	HCl	0.02	kg
	NaCl	0.24	kg
	EDTA	0.02	kg
	CTAB	0.08	kg
	chloroform	4.00	L
	isoamyl alcohol	0.17	L
	isopropanol	4.17	L
	70% ethanol	2.92	L
	electricity	18.00	kWh
	wood pellets	252.33	dry kg
Output	DNA	1.00	kg
	wastewater	98.01	kg
Sodium iodate			
Input	$NaClO_3$	0.49	kg
	Iodine	0.64	kg
	Water	0.33	kg
	Nitric acid	0.05	kg
	NaOH	0.24	kg
Output	$NaIO_3$	1.00	kg
	Wastewater	2.86	kg

Supplementary references

1. Wang, D. *et al.* Transformation of Biomass DNA into Biodegradable Materials from Gels to Plastics for Reducing Petrochemical Consumption. *J. Am. Chem. Soc.* **142**, 10114-10124 (2020).

2. Han, J., Guo, Y., Wang, H., Zhang, K. & Yang, D. Sustainable Bioplastic Made from Biomass DNA and Ionomers. *J. Am. Chem. Soc.* **143**, 19486-19497 (2021).

3. Duraj-Thatte, A. M. et al. Water-Processable, Biodegradable and Coatable Aquaplastic from Engineered Biofilms. *Nat. Chem. Biol.* **17**, 732-738 (2021).

4. Qiu. Y. et. al, Coassembly of Hybrid Microscale Biomatter for Robust, Water-Processable, and Sustainable Bioplastics. *Sci. Adv.* **11**, eadr1596 (2025).

5. Wang, J., Emmerich, L., Wu, J., Vana, P. & Zhang, K. Hydroplastic Polymers as Eco-Friendly Hydrosetting Plastics. *Nat. Sustain.* **4**, 877-883 (2021).

6. Yuan, Y. & Solin, N., Water Processable Bioplastic Films from Functionalized Protein Fibrils. *Adv. Mater. Interfaces* **9**, 2200926 (2022).

7. Young's Modulus of Common Polymers and Plastics, Materials Data Resources, Matemake, https://matmake.com/properties/youngs-modulus-of-polymers-and-plastics.html. Access date 20 April 2025.

8. ISO. *ISO 14040:2006 Environmental management - Life cycle assessment - Principles and framework*. https://www.iso.org/standard/37456.html (2006).

9. Wernet, G. *et al.* The ecoinvent database version 3 (part I): overview and methodology. *Int. J. Life Cycle Assess.* **21**, 1218–1230 (2016).

10. Santos, M., Teixeira, J. & Rodrigues, A. Production of dextransucrase, dextran and fructose from sucrose using Leuconostoc mesenteroides NRRL B512(f). *Biochem. Eng. J.* **4**, 177–188 (2000).

11. Díaz-Montes, E. Dextran: Sources, Structures, and Properties. *Polysaccharides* **2**, 554–565 (2021).

12. Moosavi-Nasab, M., Gavahian, M., Yousefi, A. R. & Askari, H. Fermentative Production of Dextran using Food Industry Wastes. *World Acad. Sci. Eng. Technol.* **68**, 875–877 (2010).

13. Kareem, A. J. & Salman, J. A. S. Production of Dextran from Locally Lactobacillus Spp. Isolates. *Reports Biochem. Mol. Biol.* **8**, 278–286 (2019).

14. Allen, G. C., Flores-Vergara, M. A., Krasynanski, S., Kumar, S. & Thompson, W. F. A modified protocol for rapid DNA isolation from plant tissues using cetyltrimethylammonium bromide. *Nat. Protoc.* **1**, 2320–2325 (2006).

15. Reads, C. Economical and Rapid Method for Extracting Cotton Genomic DNA. *J. Cotton Sci.* **4**, 193–201 (2016).

16. Bourguignon, J., Sion, M.-X. & Moreau, M. Preparation of tris (hydroxymethyl) aminomethane. US Patent 4,233,245 (1980).

17. American Chemistry Society. Cetyltrimethylammonium bromide. (2024).

18. Liu, Q. & Song, Y. A production method of Sodium periodate. (2013).

19. Bare, J. TRACI 2.0: The tool for the reduction and assessment of chemical and other environmental impacts 2.0. *Clean Technol. Environ. Policy* **13**, 687–696 (2011).

20. IPCC. *Climate Change 2022: Mitigation of Climate Change. Contribution of Working Group III to the Sixth Assessment Report of the Intergovernmental Panel on Climate Change*. (2022).

21. S. Zamenhof, Preparation and assay of deoxyribonucleic acid from animal tissue. *Methods in Enzymology* **3**, 696–704 (1957).
