# Supplementary material for: Sustainable DNA-polysaccharide hydrogels as recyclable bioplastics
Source: Nat Commun. 2025 Aug 12;16:7467. doi: 10.1038/s41467-025-62682-1 (PMC12344013; doi:10.1038/s41467-025-62682-1)
Supplement: Supplementary file 2 — Description of Additional Supplementary Files [file 41467_2025_62682_MOESM2_ESM.pdf]

Description of Additional Supplementary Files

File name: Supplementary Movie 1
Description: Represents the softening/swelling state of the bioplastics in glycerol after 7 days.

File name: Supplementary Movie 2
Description: Represents the softening/swelling state of the bioplastics in formamide after 7 days.
