# Supplementary material for: Sustainable DNA-polysaccharide hydrogels as recyclable bioplastics
Source: Nat Commun. 2025 Aug 12;16:7467. doi: 10.1038/s41467-025-62682-1 (PMC12344013; doi:10.1038/s41467-025-62682-1)
Supplement: Supplementary file 5 — Reporting Summary [file 41467_2025_62682_MOESM5_ESM.pdf]

nature portfolio

Corresponding author(s): Xi Chen, Itamar Willner, Yuwei Hu

Last updated by author(s): Jul 15, 2025

# Reporting Summary

Nature Portfolio wishes to improve the reproducibility of the work that we publish. This form provides structure for consistency and transparency in reporting. For further information on Nature Portfolio policies, see our Editorial Policies and the Editorial Policy Checklist.

## Statistics

For all statistical analyses, confirm that the following items are present in the figure legend, table legend, main text, or Methods section.

| n/a | Confirmed | |
|---|---|---|
| ☐ | ☒ | The exact sample size ($n$) for each experimental group/condition, given as a discrete number and unit of measurement |
| ☐ | ☒ | A statement on whether measurements were taken from distinct samples or whether the same sample was measured repeatedly |
| ☒ | ☐ | The statistical test(s) used AND whether they are one- or two-sided<br>*Only common tests should be described solely by name; describe more complex techniques in the Methods section.* |
| ☒ | ☐ | A description of all covariates tested |
| ☒ | ☐ | A description of any assumptions or corrections, such as tests of normality and adjustment for multiple comparisons |
| ☐ | ☒ | A full description of the statistical parameters including central tendency (e.g. means) or other basic estimates (e.g. regression coefficient) AND variation (e.g. standard deviation) or associated estimates of uncertainty (e.g. confidence intervals) |
| ☒ | ☐ | For null hypothesis testing, the test statistic (e.g. $F$, $t$, $r$) with confidence intervals, effect sizes, degrees of freedom and $P$ value noted<br>*Give P values as exact values whenever suitable.* |
| ☒ | ☐ | For Bayesian analysis, information on the choice of priors and Markov chain Monte Carlo settings |
| ☒ | ☐ | For hierarchical and complex designs, identification of the appropriate level for tests and full reporting of outcomes |
| ☒ | ☐ | Estimates of effect sizes (e.g. Cohen's $d$, Pearson's $r$), indicating how they were calculated |

*Our web collection on statistics for biologists contains articles on many of the points above.*

## Software and code

Policy information about availability of computer code

| | |
|---|---|
| Data collection | The life cycle inventory (LCI) data of the upstream burdens of producing materials, chemicals, electricity, fuels, and water were collected from the ecoinvent database and literature. The environmental impacts of commercialized plastics were based on the ecoinvent database, including PS, PET, PVC, LDPE, HDPE, PP, and PLA. (Wernet, G. et al. The ecoinvent database version 3 (part I): overview and methodology. Int. J. Life Cycle Assess. 21, 1218–1230 (2016).) Other data were collected with commercial equipments and softwares shown in Methods section. |
| Data analysis | The LCA was conducted following the ISO standard series 14040 with the software OpenLCA 2.0. Data was analyzed and graphed by OriginPro 2017, Adobe Illustrator 2024, etc. |

For manuscripts utilizing custom algorithms or software that are central to the research but not yet described in published literature, software must be made available to editors and reviewers. We strongly encourage code deposition in a community repository (e.g. GitHub). See the Nature Portfolio guidelines for submitting code & software for further information.

## Data

Policy information about availability of data

All manuscripts must include a data availability statement. This statement should provide the following information, where applicable:

- Accession codes, unique identifiers, or web links for publicly available datasets
- A description of any restrictions on data availability
- For clinical datasets or third party data, please ensure that the statement adheres to our policy

The data generated in this study are provided in the main text and Supplementary information. Data is available from the corresponding authors on request. The 1H

NMR data are available at https://doi.org/10.5281/zenodo.15878478. Other source data are provided with this paper. The life cycle inventory (LCI) data of the upstream burdens of producing materials, chemicals, electricity, fuels, and water are collected from the ecoinvent database and literature.

## Research involving human participants, their data, or biological material

Policy information about studies with human participants or human data. See also policy information about sex, gender (identity/presentation), and sexual orientation and race, ethnicity and racism.

| | |
|---|---|
| Reporting on sex and gender | Not Applicable. |
| Reporting on race, ethnicity, or other socially relevant groupings | Not Applicable. |
| Population characteristics | Not Applicable. |
| Recruitment | Not Applicable. |
| Ethics oversight | Not Applicable. |

Note that full information on the approval of the study protocol must also be provided in the manuscript.

# Field-specific reporting

Please select the one below that is the best fit for your research. If you are not sure, read the appropriate sections before making your selection.

[x] Life sciences [ ] Behavioural & social sciences [ ] Ecological, evolutionary & environmental sciences

For a reference copy of the document with all sections, see nature.com/documents/nr-reporting-summary-flat.pdf

# Life sciences study design

All studies must disclose on these points even when the disclosure is negative.

| | |
|---|---|
| Sample size | A minimum of three samples were used and analyzed. |
| Data exclusions | No data was excluded. |
| Replication | Experiments were replicated a minimum of three times. |
| Randomization | For the in vivo test, mice were randomly divided into two groups of five: a control group and a bioplastic treatment group. |
| Blinding | Blinding was not applicable for this study. |

# Reporting for specific materials, systems and methods

We require information from authors about some types of materials, experimental systems and methods used in many studies. Here, indicate whether each material, system or method listed is relevant to your study. If you are not sure if a list item applies to your research, read the appropriate section before selecting a response.

### Materials & experimental systems

| n/a | Involved in the study | |
|---|---|---|
| x | | Antibodies |
| | x | Eukaryotic cell lines |
| x | | Palaeontology and archaeology |
| | x | Animals and other organisms |
| x | | Clinical data |
| x | | Dual use research of concern |
| x | | Plants |

### Methods

| n/a | Involved in the study | |
|---|---|---|
| x | | ChIP-seq |
| x | | Flow cytometry |
| x | | MRI-based neuroimaging |

# Eukaryotic cell lines

Policy information about cell lines and Sex and Gender in Research

| | |
|---|---|
| Cell line source(s) | Primary human dermal fibroblast cells (HDF, PCS-201-012) were purchased from the American Type Culture Collection (ATCC). No cell lines were established from primary cells. |
| Authentication | Primary human dermal fibroblast cell lines are commercially available and have been tested or authenticated by the manufacturers using standard techniques. https://www.atcc.org/products/pcs-201-012 |
| Mycoplasma contamination | Mycoplasma contamination was not applicable in this study. |
| Commonly misidentified lines (See ICLAC register) | No commonly misidentified cell lines were used in this study. |

# Animals and other research organisms

Policy information about studies involving animals; ARRIVE guidelines recommended for reporting animal research, and Sex and Gender in Research

| | |
|---|---|
| Laboratory animals | Female BALB/c mice (18-22 g) between 7 and 8 weeks of age and female C57BL/6 mice (18-22 g) between 8 and 9 weeks of age were obtained from InVivos Pte Ltd, Singapore. The mice were kept at 22 °C with 50% humidity and on a 12-hour light/dark cycle with access to food and water ad libitum. |
| Wild animals | No wild animal was involved in this study. |
| Reporting on sex | No report conclusively determined that the findings apply exclusively to one sex. |
| Field-collected samples | This study did not involve any field-collected samples. |
| Ethics oversight | All animal procedures received approval from the Institutional Animal Care and Use Committee, Biological Resource Center, Singapore. |

Note that full information on the approval of the study protocol must also be provided in the manuscript.

# Plants

| | |
|---|---|
| Seed stocks | Not Applicable. |
| Novel plant genotypes | Not Applicable. |
| Authentication | Not Applicable. |
